# Propidium Monoazide Integrated with qPCR Enables the Detection and Enumeration of Infectious Enteric RNA and DNA Viruses in Clam and Fermented Sausages

**DOI:** 10.3389/fmicb.2016.02008

**Published:** 2016-12-15

**Authors:** Narciso M. Quijada, Gislaine Fongaro, Célia R. M. Barardi, Marta Hernández, David Rodríguez-Lázaro

**Affiliations:** ^1^Laboratory of Molecular Biology and Microbiology, Instituto Tecnológico Agrario de Castilla y LeónValladolid, Spain; ^2^Laboratório de Virologia Aplicada, Departamento de Microbiologia, Imunologia e Parasitologia, Universidade Federal de Santa CatarinaFlorianópolis, Brazil; ^3^Departamento de Ingeniería Agrícola y Forestal, Tecnología de los Alimentos, Escuela Técnica Superior de Ingenierías Agrarias, Universidad de ValladolidPalencia, Spain; ^4^Division of Microbiology, Department of Biotechnology and Food Science, Universidad de BurgosBurgos, Spain

**Keywords:** enteric viruses, infectivity prediction, PMA-PCR, clam, fermented sausage

## Abstract

The increase of foodborne viral outbreaks highlights the need for a rapid and sensitive method for the prediction of viral infectivity in food samples. This study assesses the use of propidium monoazide (PMA) coupled with real-time PCR methods (RT-qPCR or qPCR for RNA or DNA viruses, respectively) in the determination of viral infectivity in complex animal-related food matrices. Clam and Spanish fermented sausage (“chorizo”) samples were spiked with infectious and heat-inactivated human adenovirus-2 (HAdV-2) and mengovirus (vMC_0_). PMA-qPCR/RT-qPCR discriminated infective virus particles, with significant reductions (>2.7 log_10_ or 99.7%). Additionally, infectious HAdV-2 and vMC_0_ were quantified by plaque assay (in plaque forming units, PFU), and compared with those in virus genomes copies (GCs) quantified by PMA-qPCR/RT-qPCR. A consistent correlation (*R*^2^ > 0.92) was showed between PFU and GCs along serial 10-fold dilutions in both DNA and RNA virus and in both food matrices. This study shows the use of PMA coupled to qPCR/RT-qPCR as a promising alternative for prediction of viral infectivity in food samples in comparison to more expensive and time-consuming methods and for those viruses that are not able to grow under available cell culture techniques.

## Introduction

Food and food related environments are a major source of viral transmission to humans (Koopmans et al., 2002; Rodríguez-Lázaro et al., 2012). Several viruses, particularly noroviruses (NoV), hepatitis A virus (HAV), and hepatitis E viruses (HEV) are related to foodborne outbreaks and their incidence has increased considerably during the last years becoming a serious and widespread global public health threat (Rodríguez-Lázaro et al., 2009). Human NoV are responsible of more than five millions of gastroenteritis illnesses in USA annually, which represents more than 50% of the infectious cases (Scallan et al., 2011). These agents are recognized as major causes of foodborne illnesses, which can vary from gastroenteritis to hepatitis, paralysis or aseptic meningitis (Bosch et al., 2011).

Food-borne viruses can be present in different food matrices (Croci et al., 2008; Greening and Hewitt, 2008), although minimally processed food products, such as shellfish or fresh produced, are the most commonly related with food-borne viral outbreaks (Pintó et al., 2009). Shellfish have been considered as a major source of food-borne viruses due to their filter-feeding mechanism that can concentrate virus from polluted waters (Carter, 2005). Pork meat products are also a significant route of zoonotic transmission, as enteric viruses can infect humans from the consumption of contaminated raw or undercooked pork meat (Martínez-Martínez et al., 2011).

The increase in foodborne viral outbreaks highlights the need for rapid, sensitive, and specific methods for food safety monitoring, enabling the detection and quantification of viable foodborne viruses (Elizaquível et al., 2014). Traditional cell culture methods are laborious and time consuming, and many of the major enteric viruses cannot be or are difficult to adapt to conventional *in vitro* cell lines. The use of nucleic acid-based methods such as polymerase chain reaction (PCR) and real-time PCR (qPCR or RT-qPCR for DNA or RNA viruses, respectively) has become an alternative (Rodríguez-Lázaro et al., 2007). Nevertheless PCR-related methods are not able to discriminate between infectious and non-infectious viral particles which can drive to an overestimation of the target viruses with potential implication in public health and consequently positive results should be taken with precautions (Choi and Jiang, 2005; Hamza et al., 2011).

Coupling propidium monoazide (PMA) with PCR, qPCR or RT-qPCR appears as a promising alternative to overcome this issue. PMA is a nucleic acid intercalating dye that only crosses damaged lipid membrane barriers. Once inside, PMA binds and covalently crosslinks DNA/RNA after exposure to strong visible light, interfering DNA amplification. The rest of genomes, with the entire lipid membrane barrier, are not affected by PMA and can be amplified with PCR (Nogva et al., 2003; Nocker et al., 2006; Fittipaldi et al., 2012).

This study assesses the use of PMA coupled to qPCR or RT-qPCR in order to distinguish between infectious and non-infectious viruses in two different food matrices: clam and Spanish fermented pork sausage (“chorizo”). “Chorizo” is the most popular Spanish dry fermented sausage with more than 20 varieties described. The main ingredients are lean pork, pork fat, salt and spices (garlic, Spanish paprika, *etc*.), but the composition may vary depending on its geographical region. The raw sausage is then smoked or not and stuffed into natural or artificial casings and ripened at low temperature (12–24°C) and relatively high moisture (from 65 to 96%; González and Díez, 2002; De las Rivas et al., 2008). When the ripening process is over, “chorizo” is consumed raw. We artificially seeded the food samples with adenovirus-2 (HAdV-2) and mengovirus (vMC_0_) as virus surrogates. These viruses were chosen as they represent DNA and RNA enteric viruses, respectively: mengovirus (vMC_0_) has been used as a surrogate for several non-culturable enteric RNA viruses such as HAV and HEV, and adenoviruses have been used as bioindicators in different studies (De Motes et al., 2004; Hundesa et al., 2006).

## Materials and Methods

### Enteric Virus Production and Quantification

Mengovirus (vMC_0_) and Human adenovirus type 2 (HAdV-2) stocks were cultured in HeLa and A549 cells lines, respectively. Infected cells were freeze–thaw during three cycles after 3–5 days post-infection and cell debris was discarded after centrifugation at 600 × *g* for 30 min. Infectious viruses were quantified by plaque assay as previously described by Borden et al. (1970) and Cromeans et al. (2008). Plaques were counted after incubation at 37°C during 5–7 days.

### Food Samples

Clam and “chorizo” samples were purchased from a local supermarket. For viral analysis, 20 g of each food matrix was prepared using either a pool of clam’s digestive glands in duplicate or a pool of six parts from three different “chorizos,” for clam and “chorizo” samples, respectively. The samples were then clarified and concentrated using a glycine buffer method coupled with polyethylene glycol precipitation, and the viral particles were eluted using a glycine buffer (pH 9.5) and further concentrated with PEG 6000 precipitation (Fongaro et al., 2014). After centrifugation, supernatant was discarded and the resulting pellet was suspended in 2.0 mL of 0.1 M phosphate buffer (pH 7.2; from now on called “processed sample”).

### Artificial Contamination of Food Samples

Processed clam and “chorizo” samples were artificially contaminated with vMC_0_ 6 × 10^5^ plaque forming unity per mL (PFU mL^-1^) and HAdV-2 8 × 10^7^ PFU mL^-1^. Clam and “chorizo” processed samples were previously treated with 10 U/mL penicillin, 10 μg/mL streptomycin and 0.025 μg/mL amphotericin B and inoculated with the virus suspension (0.25 mL) at a non-cytotoxic dilution, in triplicate, in the respective cells for plaque assay. Plaques were counted after incubation at 37°C during 5–7 days.

For spiked samples, the following scenarios were performed: concentrated food samples spiked with viral fluids containing infectious HAdV-2 and vMC_0_ viruses (i) with and (ii) without addition of PMA and concentrated food samples spiked with viral fluids containing thermally inactivated HAdV-2 and vMC_0_ viruses (viral suspensions in a water bath at 95°C for 10 min; iii) with and (iv) without addition of PMA.

### PMA Treatment and Nucleic Acids Extraction

One-hundred microliters of processed samples were mixed with PMA (50 μM of final concentration -Fongaro et al., 2016; Sigma-Aldrich), and samples were incubated in a dark room at 25°C for 10 min under rotation at 200 rpm, to allow reagent penetration. Treated samples were then exposed to 40 W LED light at 460 nm wavelength for 15 min at room temperature using a photo-activation system [Led-Active Blue (PhAST Blue, Geniul)]. After the photo-activation, a rinse step was performed and nucleic acids were extracted using the DNA and RNA QIAamp Viral Mini Kit (Qiagen, Hilden, Germany), according to the manufacturer’s instructions.

### qPCR and RT-qPCR Assays

qPCR (DNA viruses) or RT-qPCR (RNA viruses) were performed for viral detection using a LightCycler 2.0 instrument (Roche Diagnostics) with the conditions previously described for HAdV-2 (Hernroth et al., 2002) and vMC_0_ (Costafreda et al., 2006), including an internal amplification control (Diez-Valcarce et al., 2012). Each sample was analyzed in duplicate. For each PCR run, four serial standard dilutions were used in triplicate for each assay, and the genome copies (GC) were calculated. Ultrapure water was used as the non-template control for each assay.

### Statistical Analyses

Results were evaluated statistically using one-way analysis of variance (ANOVA) to evaluate differences between the groups (95% confidence level), followed by Bonferroni’s Multiple Comparison Test to evaluate the differences in greater depth. Pearson test was applied for correlation analysis. The mean equivalence of PFU units (by plaque assay) versus GC (by PMA-qPCR/RT-qPCR) was tested by Linear Regression test. All statistical analyses were performed in GraphPad Prism 5.0 (USA) and the critical *p*-value for the test was set at <0.05.

## Results

### Determination of Virus Surrogates Infectivity in Food Samples Using PMA

In order to assess the interference produced by PMA, food samples were firstly spiked with infectious virus particles and then treated (or not) with PMA prior to real-time PCR (qPCR for HAdV-2 and RT-qPCR for vMC_0_). No statistically significant (*p* > 0.05) differences were observed between PMA-treated and PMA-non-treated samples spiked with identical amounts of infective viruses. Differences were below 1 log unit regardless of the type of food sample assayed: 0.42 ± 0.14 and 0.44 ± 0.17 for HAdV-2 and 0.31 ± 0.18 and 0.33 ± 0.14 for vMC_0_ in clam and “chorizo” samples, respectively.

**FIGURE 1:**
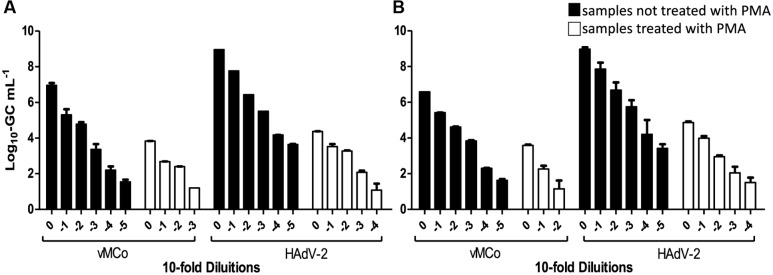
**Detection of thermally inactivated vMCo and HAdV-2 with and without PMA treatment in** **(A)** clam and **(B)** “chorizo” samples. White and black bars represent whether the samples were treated or not with PMA, respectively.

**FIGURE 2:**
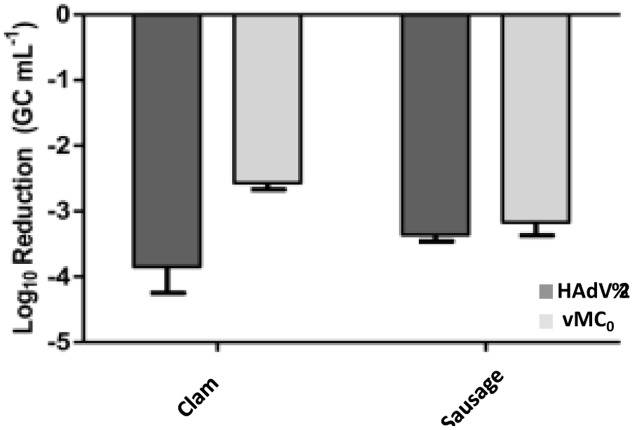
**Mean virus inactivation observed in food samples treated with PMA.** Dark and pale gray bars represent HAdV-2 and vMC_0_, respectively.

Furthermore, clam and “chorizo” samples were spiked with decreasing amounts of thermally inactivated viral particles (HAdV-2 and vMC_0_) that were submitted (or not) to a treatment with PMA prior to real-time PCR (qPCR for HAdV-2 and RT-qPCR vMC_0_) and the ability of the method for distinguishing between infectious and inactivated viruses was evaluated. Results are shown in **Figure 1**. Significant reductions of virus genome equivalents (GCs; *p* < 0.05) were observed in samples treated with PMA prior to real-time PCR, where the mean values were 3.6 ± 0.5 (log_10_ units) and 3.2 ± 0.6 for HAdV-2 and 2.7 ± 0.4 and 3.3 ± 0.1 for vMc_0_ in clam and “chorizo” samples, respectively (**Figure 2**). It represents a mean PCR derived signal reduction of 99.96 and 99.92% for HAdV-2 and 99.70 and 99.93% for vMc_0_ in clam and “chorizo” samples, respectively.

### Equivalence of PFU vs. GC

**FIGURE 3:**
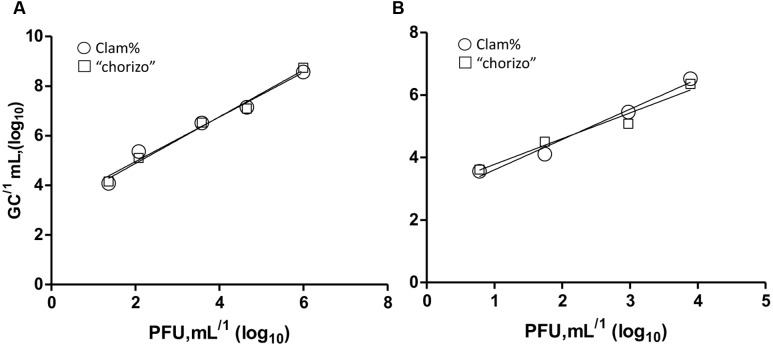
**Mean equivalences correlation of PFU and GCs in 10-fold dilutions of HAdV-2** **(A)** and vMC0 **(B)**. Balls and squares represent clam and “chorizo” samples, respectively. The *R*^2^ values were 0.92 and 0.99 for HAdV-2 and 0.96 and 0.96 for vMC_o_ in clam and “chorizo” samples, respectively.

Infectious HAdV-2 and vMC_0_ were quantified by plaque assay (PFUs) and the results were compared to those obtained in virus GCs quantified by PMA-qPCR/RT-qPCR. The mean equivalences were close to 3 log_10_ units regardless the type of food samples assayed: 2.8 ± 0.3 (log_10_ units) and 2.8 ± 0.4 for HAdV-2 and 2.6 ± 0.1 and 2.7 ± 0.4 for vMC_0_ in clam and “chorizo” samples, respectively. These equivalences were calculated and confirmed along 10-fold dilutions of PFU units by statistical testing [Linear Regression, GraphPad Prism version 5.0 (USA)]. The *R*^2^ values obtained ranged from 0.92 to 0.99, demonstrating an excellent linearity regardless the dilution of the PFU units and food samples tested (**Figure 3**).

## Discussion

Food-borne diseases are one of the major priorities of the World Health Organization (WHO) as they are widespread and their incidence has increased during the last years (Elizaquível et al., 2014). Enteric viruses, such as human NoV, hepatitis A and E viruses, enteroviruses, rotaviruses, or astroviruses are present in food products and those that are consumed raw or undercooked are commonly related with viral foodborne outbreaks. These products include fruits, vegetables, shellfish (oysters, clams and mussels) and pig-derived products such as fermented meat products consumed raw (Le Guyader et al., 2006; Parshionikar et al., 2010; Sanchez et al., 2012). Hence, the development of methods that allow the rapid and sensitive detection and quantification of enteric viruses in food products are of great value to the food industry (Perelle et al., 2007). Detection of enteric viruses based only in genome detection does not provide an accurate analysis of viral load and cannot determine the infectivity potential. Viral infectivity studies include cell culture methods in order to demonstrate cytopathic effect. Such experiments can be laborious, and many viruses do not produce cytopathic effects (Rodríguez et al., 2009). Several viability staining-PCR strategies have been used in detection foodborne pathogens using different dyes such as PMA or ethidium monoazide (EMA). These dyes penetrate only in capside-compromised viruses. However, the applicability of this viability-PCR strategy must be tested for each type of food matrix due to composition complexity of foods, and the use of PMA as dye for sample pretreatment previous to the qPCR has been described as more suitable (Elizaquível et al., 2014).

Although coupling PMA with qPCR or RT-qPCR has been successfully applied to discriminate infectious and inactivated virus particles from viral suspension, river water, vegetables, swine raw manure, swine effluent from anaerobic biodigesters and biofertilized soil (Parshionikar et al., 2010; Sanchez et al., 2012; Coudray-Meunier et al., 2013; Moreno et al., 2015; Fongaro et al., 2016) little is known about the applicability of this technique in complex animal origin foods. In this study we chose two different food matrices: clam and Spanish fermented sausage (“chorizo”). Clam is a widely consumed product and they are known to concentrate virus from polluted water due to its filtering-feeding mechanism (Carter, 2005). “Chorizo” is a pork sausage made of combination of pork lean and fat with several spices (including Spanish paprika) and it is consumed raw after ripening.

The results of this study show that coupling PMA with qPCR or RT-qPCR successfully discriminates among infective and inactivated HAdV-2 and vMC_0_ in clam and “chorizo” samples. This is the first study that assesses the possible usage of PMA coupled with qPCR/RT-qPCR in the determination of the infectivity of viral particles on food samples from animal origin. PMA activity was not inhibited by the processing of the two complex food matrices tested and did not interfere significantly in the genome quantification by qPCR/RT-qPCR. The hit of this study is the successful application of the method over complex food matrices, highlighting the use of PMA coupled with qPCR/RT-qPCR as a promising alternative for enteric virus determination in food safety studies. Noteworthy, a consistent correlation (*R*^2^ > 0.92) was observed along serial 10-fold dilutions in both DNA and RNA virus and in both clam and “chorizo” samples, even though a complete reduction of the signal for thermally inactivated virus particles was not obtained.

## Conclusion

The combination of PMA with qPCR/RT-qPCR is a promising alternative for enteric virus investigation in food safety, particularly regarding to non-cultivable viruses, that allows the discrimination between damaged and undamaged virus particles in animal-related food products.

## Author Contributions

NQ and GF performed the experiments and contributed in the preparation of the first draft of the manuscript. MH collaborated in the definition of the experimental part and contributed in the revision of the experiments and preparation of the final version of the manuscript. CB contributed in the preparation of the final version of the manuscript, and DR-L defined the experimental approach, revised the results and led the preparation of the manuscript.

## Conflict of Interest Statement

The authors declare that the research was conducted in the absence of any commercial or financial relationships that could be construed as a potential conflict of interest.
